# Effect of artificial gravity on calcaneal bone marrow adipose tissue and mineral content in female and male participants in 60 days of bed rest

**DOI:** 10.1113/EP091495

**Published:** 2025-03-23

**Authors:** Tammy Liu, Gerd Melkus, Tim Ramsay, Alain Berthiaume, Gabriele Armbrecht, Guy Trudel

**Affiliations:** ^1^ Bone and Joint Research Laboratory Ottawa Hospital Research Institute Ottawa Ontario Canada; ^2^ Department of Radiology University of Ottawa Ottawa Ontario Canada; ^3^ Department of Radiology, Centre for Muscle and Bone Research Charité Universitätsmedizin Berlin Berlin Germany; ^4^ Department of Medicine, Division of Physiatry, Faculty of Medicine University of Ottawa Ottawa Ontario Canada; ^5^ Department of Cellular and Molecular Medicine, Faculty of Medicine University of Ottawa Ottawa Ontario Canada

**Keywords:** bed rest study, bone marrow densitometry, calcaneal fat fraction, magnetic resonance imaging, marrow adipose tissue

## Abstract

Modulation of bone marrow adipose tissue (BMAT) with prolonged inactivity was reported in haemopoietic but not in non‐haemopoietic bones. This prospective randomized controlled trial submitted 16 men and 8 women to 60 days of 6° head‐down‐tilt bed rest. They were assigned to control, continuous or intermittent artificial gravity (AG) interventions. The AG consisted of daily centrifugation at 2*g* for 30 min. The serial foot pain questionnaire, MRI and dual‐energy X‐ray absorptiometry of the calcaneus were performed at baseline, during bed rest and at reambulation. At baseline, all groups had comparable calcaneal BMAT (*P* = 0.581) and bone mineral density (BMD) (*P* = 0.574). After bed rest, 83% of participants reported foot pain. Calcaneal BMAT was not significantly modulated after 60 days of bed rest (control, +0.2% ± 0.8%; continuous AG, +0.5% ± 1.1%; and intermittent AG, +0.1% ± 1.5%; *P* = 0.368). Calcaneal BMD was reduced at reambulation days 3 and 11 after 60 days of bed rest (−0.05 ± 0.06 and −0.06 ± 0.12 g/cm^2^, respectively; *P* = 0.008 and *P* = 0.020). The AG interventions did not significantly alter calcaneal BMAT or BMD. Sex‐based analyses demonstrated calcaneal BMD loss in men but not in women. Calcaneal BMAT and BMD were inversely correlated in women and in men (Spearman's ρ, −0.40 and −0.28, respectively; both *P* = 0.020). Sixty days of bed rest caused foot pain and calcaneal demineralization not rescued by AG interventions. Although inversely correlated with BMD, calcaneal BMAT was not statistically increased by 60 days of head‐down‐tilt bed rest, possibly owing to a ceiling effect, and no bone marrow reconversion was measured at reambulation. These results have clinical relevance when returning to activities after prolonged bed rest or returning from space.

## Abstract (highlights)

- **What is the central question of this study?**
  What is the efficacy of artificial gravity to prevent increased calcaneal bone marrow adipose tissue and reduced bone mineral content during 60 days of bed rest?
- **What is the main finding and its importance?**
  Calcaneal bone mineral density decreased with 60 days of bed rest, whereas calcaneal bone marrow adipose tissue was unchanged. Two different artificial gravity protocols did not rescue calcaneal bone loss or prevent foot pain. These findings suggest searching for new countermeasures against the deleterious effects of prolonged bed rest to prevent foot bone loss from bed rest and in space.

## 1 INTRODUCTION

Inactivity on Earth and microgravity in space eliminate the ground reaction forces as a stimulus for bone formation, particularly in the lower limbs (Man et al., 2022). Mice in microgravity showed bone loss (Blaber et al., 2013; Gerbaix et al., 2017; Maupin et al., 2019). Humans exposed to microgravity analogues or to space also experienced decreased calcaneal bone mineral density (BMD) (Shackelford et al., 2004; Wang et al., 2012). Bone marrow adipocytes are the unique adipocytes that reside in the bone marrow microenvironment (Bravenboer et al., 2020). Bone marrow adipose tissue (BMAT) as an organ responds to internal and external factors and produces adipokines to influence metabolism (Scheller & Rosen, 2014). In normal ageing, the haemopoietic marrow is converted to fatty marrow in a peripheral to central direction (Baum et al., 2018). Various clinical situations were shown to accelerate this fatty marrow conversion; increases in BMAT have been associated with osteoporosis (Di Iorgi et al., 2008; Paccou et al., 2019), inactivity (Liu et al., 2021; Trudel et al., 2012, 2009) and spaceflight (Liu et al., 2023), in addition to cardiovascular disease (Wang et al., 2018), glucose intolerance (Wan et al., 2023), hormonal changes (Beekman et al., 2022), ageing (Ambrosi et al., 2017; Beekman et al., 2022), cancer (Fairfield et al., 2021) and diet (Bredella et al., 2009; Doucette et al., 2015; Fazeli et al., 2013; Tencerova et al., 2018). Most experimental data were obtained by studying haemopoietically active bones. Investigation of BMAT in a bone, the calcaneus, not involved with haemopoiesis might contribute key new elements regarding its modulation.

The BMAT and BMD have an intimate relationship, and many studies have suggested that they are inversely correlated (Di Iorgi et al., 2008; Liu et al., 2023; Shen et al., 2007). Importantly, a tailored rehabilitation programme after prolonged inactivity must consider these bone changes, because a return to activity is accompanied by foot pain (Parry & Puthucheary, 2015), and a demineralized calcaneus is a heightened risk for Achilles tendon avulsion rupture (Matsumoto et al., 2003; Squires et al., 2001; Trudel et al., 2007; Wren et al., 2001). Microgravity analogues in which participants undergo prolonged bed rest (60 days) in the antiorthostatic position (−6°) with their lower limbs unloaded constitute a unique opportunity to study the effects of unloading on weight‐bearing bones.

Clinical interventions to prevent bone demineralization with skeletal unloading are few. Antiresorptive agents protected bone after spinal cord injury, but the effect was not sustained after discontinuation (Anastasilakis et al., 2021; Bone et al., 2011). Mechanical stimulation using exercise and whole‐body vibration have shown positive effects on BMD and BMAT (Belavy et al., 2011; Boeselt et al., 2016; Jo et al., 2021; Trudel et al., 2012). A human short‐arm centrifuge seeking to recreate a gravity force vector (artificial gravity or AG) has shown promise in preventing the effects of prolonged inactivity (Clément et al., 2016; Goswami et al., 2015; Hargens et al., 2013; Stenger et al., 2012).

We carried out serial measurements of the effects of two different AG protocols on preventing changes in foot pain [measuring calcaneus BMD using dual‐energy X‐ray absorptiometry (DXA) and BMAT using 3 T MRI] in 24 female and male participants before, during and up to 15 months after 60 days of 6° head‐down tilt (HDT) bed rest (HDTBR). Our hypothesis was that AG will prevent calcaneal BMAT accumulation, osteopenia and foot pain at reambulation in comparison to control subjects.

## 2 MATERIALS AND METHODS

### 2.1 Ethics approval

The participants provided written informed consent conforming to the *Declaration of Helsinski*. The trial was approved by the German Aerospace Center (DLR) Ethics Committee (protocol number 018143), Federal Office for Radiation Protection (number Z 5‐22464/2018‐074‐R‐G, Bundesamt für Strahlenschutz), Ottawa Health Science Network Research Ethics Board (OHSN‐REB; protocol number 20190023‐01H) and registered at the German Clinical Trials Register (DRKS‐ID: DRKS00015677).

### 2.2 Participants

**FIGURE 1:**
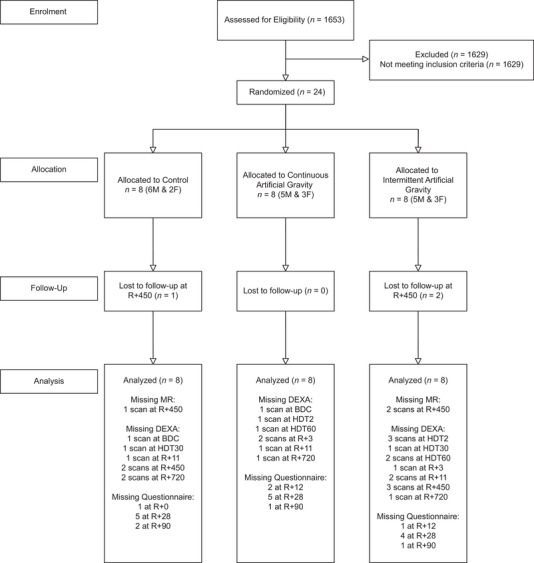
Consort flow diagram. Three participants were lost to follow‐up at R450, and 24 BMD scans were excluded owing to technical limitations of the DXA system. One participant did not respond to the pain questionnaire at R+0, 3 participants at R+12, 14 participants at R+28, and 4 participants at R+90. Abbreviations: BDC, baseline data collection; BMD, bone mineral density; DXA, dual‐energy X‐ray absorptiometry; F, female; HDTBR, head‐down‐tilt bed rest; M, male; R, reambulation.

Twenty‐four healthy participants (16 men and 8 women) were recruited from public media advertisement (Figure 1). Inclusion and exclusion criteria were extensive (Appendix). The trial involved >20 research proposals testing the effect of bed rest and of AG on multiple body systems. We investigated the effects on foot pain, calcaneal BMD and marrow adipose tissue.

### 2.3 Schedule

The study was performed at the:envihab research facility of the DLR Institute of Aerospace Medicine in Cologne, Germany. Participants completed the study in two consecutive campaigns (*n* = 12 each). Both campaigns were identical in that participants stayed for a total of 88 days: 14 days of baseline data collection (BDC), 60 days of 6° HDTBR and 14 days of reambulation (R). All participants underwent six MR scans (BDC‐12, HDT31, HDT60, R6, R90 and R450) of their right calcaneus. The planned 1‐year reambulation follow‐up (R365) was delayed to R450 owing to the coronavirus disease 2019 pandemic. The study began in March 2019 and concluded in December 2020.

### 2.4 Artificial gravity countermeasure

Participants were randomly assigned to one of three experimental groups (*n* = 8 in each group): (1) control, 6° HDTBR with no centrifugation; (2) continuous centrifugation (cAG), 6° HDTBR with supine centrifugation at +2*gz* at the feet for 30 min per day; or intermittent centrifugation (iAG), 6° HDTBR with supine centrifugation at +2*gz* at the feet for six bouts of 5 min per day (each session is separated by a 5 min rest period). The study sponsors wanted to compare the efficacy and tolerability of the two AG protocols.

### 2.5 Foot pain questionnaire

Participants serially filled questionnaires about whether they experienced foot pain within the last 24 h. Participants were asked to indicate the location, the quality, factors that provoke or palliate, whether the pain radiated, the severity and the duration. Pain data were obtained at 20 experimental time points: 12 and 1 day before bed rest; day 7, 14, 21, 28, 35, 42, 49 and 56 during bed rest; and day 0, 1, 2, 3, 4, 5, 6, 12, 28 and 90 of reambulation. We reported data on all foot pain, whether medial, plantar, posterior or heel. Pain scores range from 0 to 100, where 0 indicates the absence of pain and 100 represents the worst possible pain. Foot pain scores were averaged between the left and right feet.

### 2.6 Quantitative MRI protocol

Magnetic Resonance imaging (MRI) was performed on a Siemens 3 Tesla MRI scanner (Biograph mMR) using the following sequence: sagittal T_1_ Volumetric Interpolated Breath‐hold Examination (VIBE) with IN, OUT, FAT and WATER reconstructions (isotropic resolution 1 mm) [Repetition time (TR), 6.3 ms; Echo Time (TE)_1_, 2.46 ms (in phase); TE_2_, 3.69 ms (out of phase); field of view, 250 mm × 250 mm; matrix, 256 × 256; slice thickness, 1 mm; Echo Train Length (ETL), 2; flip angle, 10°; number of averages, 1).

### 2.7 Calcaneal bone marrow adiposity

The T_1_‐weighted sequence acquisitions were processed using in‐house software programs developed in Matlab 2014a (Mathworks, Natick, MA, USA) to create fat fraction masks. The three most central consecutive axial slices of the calcaneus were selected. A polygonal region of interest (ROI) was drawn manually over the calcaneus excluding the cortical bone using ITK‐SNAP (Yushkevich et al., 2006). The ROI was applied on the corresponding calcaneal fat fraction maps:

$$\mathrm{BMA}T_{\mathrm{DIXON}}=\frac{\mathrm{Imag}e_{\mathrm{fat}}}{\mathrm{Imag}e_{\mathrm{fat}}+\mathrm{Imag}e_{\mathrm{water}}}$$

where BMAT is the bone marrow adipose tissue in the calcaneus, Image_fat_ is the the fat image and Image_water_ is the water image. The BMAT data were obtained as the average bone marrow fat fraction from three axial slices of the calcaneus.

### 2.8 Dual‐energy X‐ray absorptiometry

Dual‐energy X‐ray absorptiometry of the calcaneus was obtained using a GE Healthcare Prodigy Pro system (GE Healthcare, WI, USA). Bone mineral density data were obtained by manually drawing a global ROI over the calcaneus at the baseline measurement. A second elliptical inner ROI was drawn within the calcaneus to include only the trabecular region at the baseline measurement. For each subsequent experimental time point, compare modus was used to copy the ROIs from baseline, using the global ROI for comparable positioning of the inner ROI. This ensured that the positioning and area of the trabecular ROI were identical. Areal bone mineral density (aBMD), bone mineral content (BMC), BMD and *T*‐Score were obtained at eight experimental time points: BDC, HDT2, HDT30, HDT60, R+3, R+11, R+450 and R+720.

### 2.9 Data and statistical analysis

Data were analysed using SPSS Statistics (v.28.0; IBM, Armonk, NY, USA). They are presented as the average with one standard deviation except where otherwise stated. We tested for the effect of campaign on calcaneal BMAT and BMD using a two‐sided Mann–Whitney *U*‐test for the cohort at all experimental time points, then at each experimental time point. Given that they were identical (Table 1), we combined data from both campaigns to analyse all participants (*n* = 24). We tested the effect of bed rest on calcaneal BMAT and BMD using two‐sided Wilcoxon signed rank tests for the cohort by comparing each time point with baseline. We also tested the effect of bed rest on calcaneal BMAT and BMD using two‐sided Wilcoxon signed rank tests in each experimental group by comparing each time point with baseline. We tested the effect of AG protocols on calcaneal BMAT and BMD using Kruskal–Wallis tests for the cohort and at each experimental time point. We tested for the effect of sex on calcaneal BMAT and BMD using two‐sided Mann–Whitney *U*‐tests for the cohort and at each experimental time point. We ran correlations between BMAT and BMD of the cohort using Spearman's ρ. We performed descriptive statistics on the foot pain data.

**TABLE 1:** Mean foot pain before, during and after 60 days of head‐down‐tilt bed rest.

Time	Cohort (*n* = 24)	Control (*n* = 8)	cAG (*n* = 8)	iAG (*n* = 8)
BDC‐12	0.0 ± 0.0	0.0 ± 0.0	0.0 ± 0.0	0.0 ± 0.0
BDC‐1	0.0 ± 0.0 (*P* = 1.00)	0.0 ± 0.0 (*P* = 1.00)	0.0 ± 0.0 (*P* = 1.00)	0.0 ± 0.0 (*P* = 1.00)
HDT7	0.08 ± 0.38 (*P* = 0.317)	0.23 ± 0.66 (*P* = 0.317)	0.0 ± 0.0 (*P* = 1.00)	0.0 ± 0.0 (*P* = 1.00)
HDT12	0.03 ± 0.13 (*P* = 0.317)	0.08 ± 0.22 (*P* = 0.317)	0.0 ± 0.0 (*P* = 1.00)	0.0 ± 0.0 (*P* = 1.00)
HDT21	0.35 ± 1.71 (*P* = 0.317)	1.05 ± 2.96 (*P* = 0.317)	0.0 ± 0.0 (*P* = 1.00)	0.0 ± 0.0 (*P* = 1.00)
HDT28	0.05 ± 0.26 (*P* = 0.317)	0.16 ± 0.44 (*P* = 0.317)	0.0 ± 0.0 (*P* = 1.00)	0.0 ± 0.0 (*P* = 1.00)
HDT35	0.13 ± 0.61 (*P* = 0.317)	0.38 ± 1.06 (*P* = 0.317)	0.0 ± 0.0 (*P* = 1.00)	0.0 ± 0.0 (*P* = 1.00)
HDT42	0.34 ± 1.18 (*P* = 0.317)	1.02 ± 1.95 (*P* = 0.180)	0.0 ± 0.0 (*P* = 1.00)	0.0 ± 0.0 (*P* = 1.00)
HDT49	0.24 ± 0.94 (*P* = 0.180)	0.72 ± 1.59 (*P* = 0.180)	0.0 ± 0.0 (*P* = 1.00)	0.0 ± 0.0 (*P* = 1.00)
HDT56	0.44 ± 1.81 (*P* = 0.180)	0.22 ± 0.62 (*P* = 0.317)	1.09 ± 3.09 (*P* = 0.317)	0.0 ± 0.0 (*P* = 1.00)
R+0	6.55 ± 9.41^a^ (*P* = 0.005)	13.23 ± 11.55^a^ (*P* = 0.043)	4.72 ± 8.65 (*P* = 0.109)	2.53 ± 4.71 (*P* = 0.180)
R+1	4.25 ± 7.86^a^ (*P* = 0.012)	4.97 ± 9.06 (*P* = 0.109)	6.44 ± 9.52 (*P* = 0.068)	1.34 ± 3.80 (*P* = 0.317)
R+2	4.79 ± 7.13^a^ (*P* = 0.005)	7.80 ± 8.92^a^ (*P* = 0.043)	4.33 ± 5.38 (*P* = 0.068)	2.25 ± 6.36 (*P* = 0.317)
R+3	3.83 ± 5.50^a^ (*P* = 0.005)	5.59 ± 6.60^a^ (*P* = 0.043)	3.36 ± 4.94 (*P* = 0.109)	2.53 ± 5.07 (*P* = 0.180)
R+4	4.20 ± 6.13^a^ (*P* = 0.003)	5.53 ± 5.97^a^ (*P* = 0.043)	3.89 ± 6.12 (*P* = 0.068)	3.19 ± 6.58 (*P* = 0.180)
R+5	2.96 ± 6.61^a^ (*P* = 0.018)	2.05 ± 5.26 (*P* = 0.180)	3.67 ± 5.90 (*P* = 0.068)	3.16 ± 8.93 (*P* = 0.317)
R+6	4.65 ± 6.96^a^ (*P* = 0.003)	4.78 ± 6.26 (*P* = 0.068)	4.11 ± 4.86^a^ (*P* = 0.043)	5.06 ± 9.78 (*P* = 0.180)
R+12	5.74 ± 5.73^a^ (*P* < 0.001)	4.59 ± 4.32^a^ (*P* = 0.043)	3.94 ± 3.76^a^ (*P* = 0.043)	8.61 ± 7.82^a^ (*P* = 0.042)
R+28	0.54 ± 1.02 (*P* = 0.102)	0.0 ± 0.0 (*P* = 1.00)	0.38 ± 0.65 (*P* = 0.317)	1.06 ± 1.47 (*P* = 0.180)
R+90	0.08 ± 0.36 (*P* = 0.317)	0.0 ± 0.0 (*P* = 1.00)	0.23 ± 0.61 (*P* = 0.317)	0.0 ± 0.0 (*P* = 1.00)

*Note*: Pain scores range from 0 to 100, where 0 indicates the absence of pain and 100 represents the worst possible pain. Abbreviations: BDC, baseline data collection; cAG, continuous artificial gravity; HDT, head‐down‐tilt; iAG, intermittent artificial gravity; R, reambulation.

^a^ *P*‐value compared with baseline average foot pain score by two‐sided Wilcoxon signed rank test.

## 3 RESULTS

All 24 participants completed the study (Figure 1). Three participants were lost to follow‐up at R450, leaving *n* = 141/144 MRI scans available for analyses. Twenty‐four BMD scans were excluded owing to technical limitations of the DXA system, leaving *n* = 165/192 BMD measures available for analyses (Figure 1). One participant did not respond to the pain questionnaire at R+0, 3 participants at R+12, 14 participants at R+28, and 4 participants are R+90, leaving *n* = 458/480 pain scores for analyses.

### 3.1 Calcaneal bone marrow adipose tissue

**FIGURE 2:**
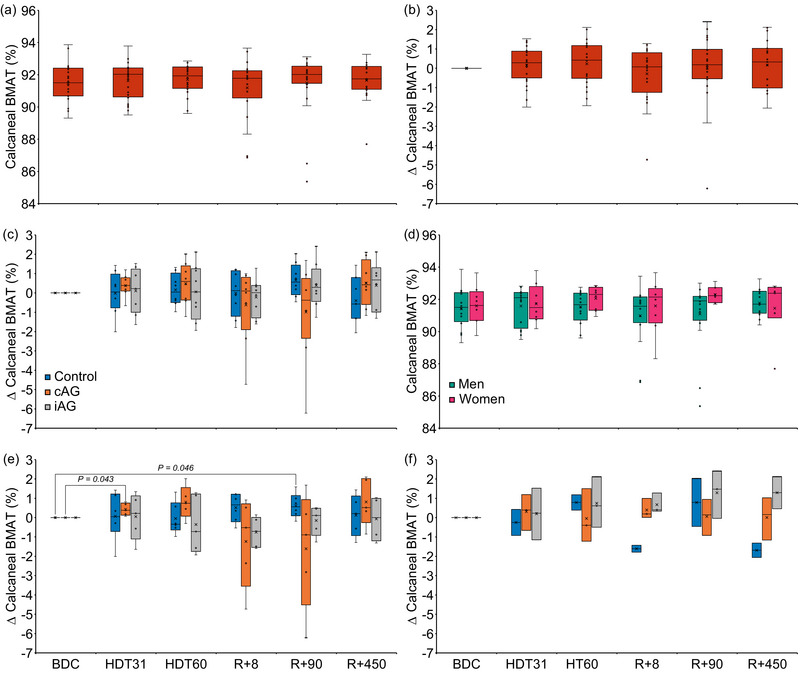
Calcaneal BMAT caused by 60 days of head‐down‐tilt bed rest. (a) BMAT in the cohort (*n* = 141). (b) Change in BMAT in the cohort (*n* = 141). (c) Effect of AG interventions on BMAT (control, *n* = 47; cAG, *n* = 48; iAG, *n* = 46). (d) Sex differences between men (*n* = 94) and women (*n* = 47). (e) Effect of AG interventions on BMAT in men (control, *n* = 35; cAG, *n* = 30; iAG, *n* = 29). (f) Effect of AG interventions on BMAT in women (control, *n* = 12; cAG, *n* = 18; iAG, *n* = 17). Neither bed rest nor AG interventions modulated calcaneal BMAT before, during and after bed rest. Abbreviations: AG, artificial gravity; BDC, baseline data collection; BMAT, bone marrow adipose tissue; cAG, continuous artificial gravity; HDT, head‐down tilt; iAG, intermittent artificial gravity; R, reambulation.

Baseline average calcaneal BMAT was 91.5% ± 1.2% (Figure 2a). Bed rest for 31 and 60 days minimally increased BMAT by 0.2% ± 1.0% (*P* = 0.304) and 0.2% ± 0.4% (*P* = 0.253), respectively (Figure 2b). Likewise, 8 and 90 days of reambulation after bed rest caused no significant differences in calcaneal BMAT (*P* = 0.648 and *P* = 0.331, respectively; Figure 2b). At 450 days of follow‐up, calcaneal BMAT was 91.7% ± 1.2%, comparable to baseline (*P* = 0.741; Figure 2a).

### 3.2 Effects of AG on BMAT

At baseline, the three experimental groups had comparable BMAT (control, 91.3% ± 1.1%; cAG, 91.3 ± 1.4%; and iAG, 91.9% ± 1.1%; *P* = 0.581). After 60 days of bed rest, calcaneal BMAT remained unchanged in all three groups (control, +0.2% ± 0.8%; cAG, +0.5% ± 1.1%; and iAG, +0.1% ± 1.5%; *P* = 0.368; Figure 2c). At follow‐up 450 days, the changes in calcaneal BMAT with AG (cAG, +0.5% ± 1.2%; and iAG, +0.4% ± 1.3%) were not statistically different from the BMAT change in controls (−0.4% ± 1.3%; *P* = 0.344; Figure 2c).

### 3.3 BMAT sex‐based analyses

At baseline, calcaneal BMAT was comparable between men and women (91.4% ± 1.2% vs. 91.6% ± 1.2%, respectively; *P* = 0.834; Figure 2d). BMAT was not significantly different between men and women at all experimental time points (*P* = 0.928, 0.192, 0.238, 0.136 and 0.856; Figure 2d). Male participants receiving cAG after 31 days of bed rest and those in the control group at 90 days of reambulation showed higher BMAT (+0.43% ± 0.32% and +0.62% ± 0.63%, respectively; *P* = 0.043 and *P* = 0.046, respectively; Figure 2e). Artificial gravity protocols cAG or iAG did not influence calcaneal BMAT in female participants during the bed rest and reambulation phases of the study (*P* = 0.593, 0.593, 1.00, 0.285, 0.109, 0.109, 0.593, 0.295, 1.00 and 0.180; Figure 2f).

### 3.4 Calcaneal bone mineral density

**FIGURE 3:**
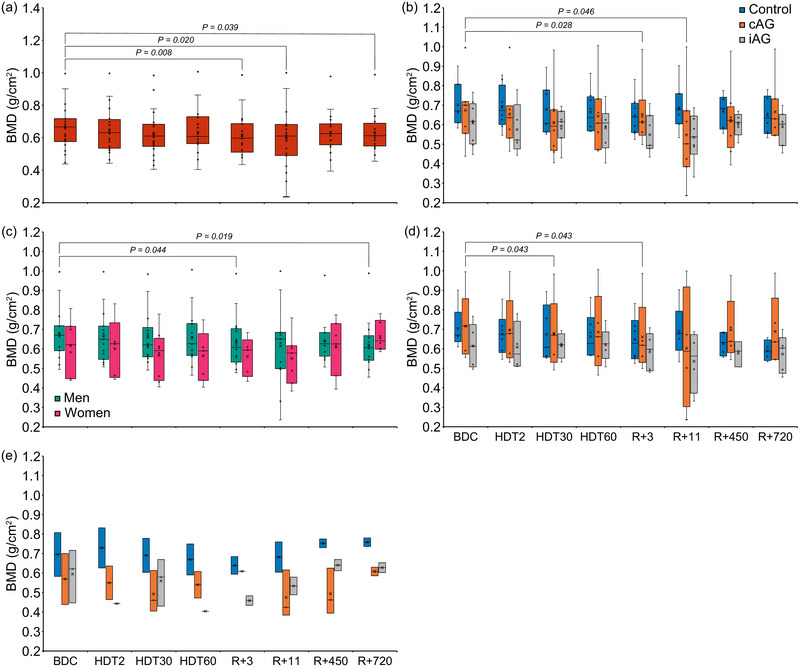
Calcaneal BMD following 60 days of head‐down‐tilt bed rest. (a) BMD in the cohort (*n* = 165). (b) Effect of AG interventions on BMD (control, *n* = 57; cAG, *n* = 57; iAG, *n* = 51). (c) Sex differences between men (*n* = 115) and women (*n* = 50). (d) Effect of AG interventions on BMD in men (control, *n* = 41; cAG, *n* = 39; iAG, *n* = 35). (e) Effect of AG interventions on BMD in women (control, *n* = 16; cAG, *n* = 19; iAG, *n* = 16). Sixty days of head‐down tilt caused significant calcaneal demineralization in the cohort and in men only (*P* = 0.044). Calcaneal bone loss was not rescued by either AG intervention (*P *> 0.05). Calcaneal BMD recovery was incomplete 2 years after 60 days of bed rest (*P* = 0.019). Abbreviations: AG, artificial gravity; BDC, baseline data collection; BMD, bone mineral density; cAG, continuous artificial gravity; HDT, head‐down tilt; iAG, intermittent artificial gravity; R, reambulation.

At baseline, the calcaneal BMD was 0.66 ± 0.14 g/cm^2^ (Figure 3a). Following 60 days of bed rest, calcaneal BMD was lower on reambulation day 3 (−0.05 ± 0.06 g/cm^2^; −7.00% ± 9.23%), reambulation day 11 (−0.06 ± 0.12 g/cm^2^; −9.76% ± 17.24%) and reambulation day 720 (−0.03 ± 0.07 g/cm^2^; −3.85% ± 10.09%) (*P* = 0.008, 0.020 and 0.039, respectively; Figure 3a).

### 3.5 Effects of AG on BMD

At baseline, all experimental groups had comparable calcaneal BMD (control, 0.70 ± 0.11 g/cm^2^; cAG, 0.67 ± 0.18 g/cm^2^; and iAG, 0.61 ± 0.11 g/cm^2^; *P* = 0.574). The participants in the cAG group showed statistically significant decreases in calcaneal BMD at reambulation days 3 (−0.06 ± 0.04 g/cm^2^; −9.01% ± 5.01%; *P* = 0.028) and 11 (−0.11 ± 0.18 g/cm^2^; −16.33% ± 25.26%; *P* = 0.046) compared with baseline (Figure 3b). Direct comparison between the three groups at every time point showed no statistically significant difference.

### 3.6 BMD sex‐based analyses

Men had lower calcaneal BMD at reambulation day 3 compared with baseline (0.63 ± 0.13 vs. 0.68 ± 0.13 g/cm^2^; −5.90% ± 9.14%; *P* = 0.044; Figure 3c). Still in men, calcaneal BMD remained lower than baseline after 2 years of reambulation (0.62 ± 0.13 g/cm^2^; −5.50% ± 6.61%; *P* = 0.019; Figure 3c). Direct comparison between the three groups at every time point in male or female participants showed no statistically significant effect of the AG interventions (Figure 3d, e).

### 3.7 Correlation between BMAT and BMD

**FIGURE 4:**
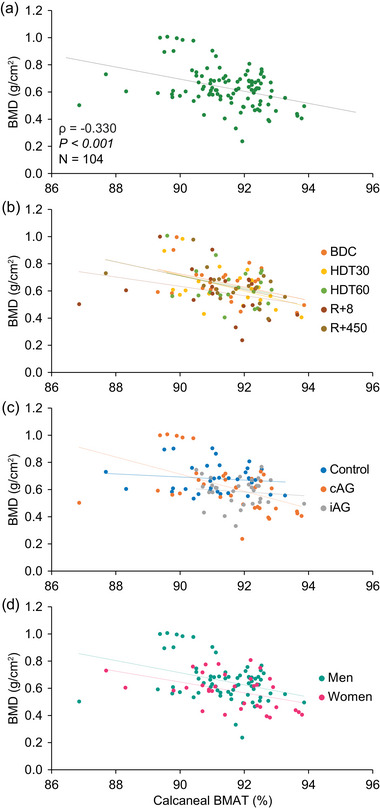
Spearman's correlation between calcaneal BMAT and BMD. (a) Correlation between BMAT and BMD (*n* = 104). (b) Correlations colour coded by experimental time point (*n* = 104). (c) Correlations colour coded by AG intervention (*n* = 104). (d) Correlations colour coded by sex (*n* = 70). Calcaneal BMAT was inversely correlated with BMD. Abbreviations: AG, artificial gravity; BDC, baseline data collection; BMAT, bone marrow adipose tissue; BMD, bone mineral density; HDT, head‐down tilt; R, reambulation.

The BMAT and BMD were inversely correlated for the cohort at all time points (ρ = −0.33; *P *< 0.001; Figure 4a). The inverse correlation was maintained at all bed rest study time points (Figure 4b). The type of intervention did not reverse the negative correlation between BMAT and BMD (Figure 4c). Sex‐based analyses also showed an inverse correlation between calcaneus BMAT and BMD in women and men (women, ρ = −0.40; men, ρ = −0.28; both *P* = 0.020; Figure 4d).

### 3.8 Foot pain

**FIGURE 5:**
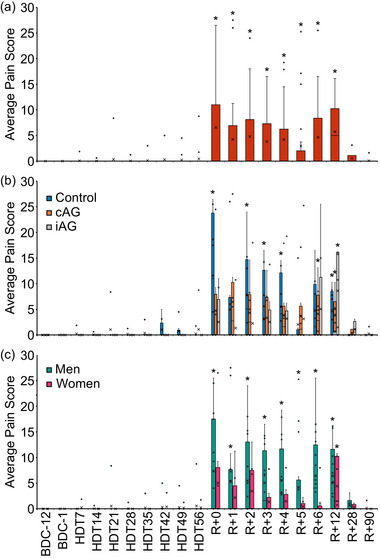
Mean foot pain scores on a scale of 0–100. (a) Mean foot pain before, during and after 60 days of head‐down‐tilt bed rest (*n* = 453). (b) Effect of AG interventions on mean foot pain (control, *n* = 150; cAG, *n* = 151; iAG, *n* = 152). (c) Sex differences in foot pain (men, *n* = 301; women, *n* = 152). In comparison to baseline, participants complained of higher levels of foot pain during the first 2 weeks of reambulation (exact *P*‐values are given in Table 1). Participants in the control group experienced higher mean foot pain at more reambulation time points than those in the AG intervention groups (exact *P*‐values are given in Table 1). At most reambulation time points, men reported higher pain scores in comparison to baseline (exact *P*‐values are given in Table 1). Abbreviations: AG, artificial gravity; BDC, baseline data collection; cAG, continuous artificial gravity; HDTBR, head‐down tilt; iAG, intermittent artificial gravity; R, reambulation.

Twenty of 24 participants (14 men and 6 women) reported foot pain during the study. Pain was experienced as early as day 7 of bed rest and persisted until 90 days after recovery, with most participants experiencing foot pain immediately upon reambulation (Table 1). Mean foot pain was statistically higher at reambulation days 0, 1, 2, 3, 4, 5, 6 and 12 compared with baseline (Table 1; Figure 5a). Foot pain was higher in the control group on reambulation days 0, 2, 3, 4 and 6, and for both cAG and iAG groups at reambulation day 12 (Table 1; Figure 5b). Mean foot pain in men was higher at reambulation days 0, 1, 2, 3, 4, 5, 6 and 12, and only at reambulation day 12 for women (Table 1; Figure 5c). There were no differences in mean foot pain between interventions at each time point. There were no sex differences in foot pain at each time point (Table 1).

## 4 DISCUSSION

This prospective, randomized controlled clinical trial identified foot pain and calcaneal demineralization with prolonged bed rest, which AG was ineffective at preventing, and quantified, for the first time, calcaneal BMAT during and after prolonged antiorthostatic bed rest.

Foot unloading in a lower limb non‐weight‐bearing cast, during bed rest or in space disrupts physiology. Astronauts carry their missions without footwear because the feet are unloaded for most of their mission (Cavanagh et al., 2010), and they report high rates of foot injury (Scheuring et al., 2009). Our results of prevalent low‐grade foot pain at reambulation in the space analogue of prolonged antiorthostatic bed rest agree with this literature. We contributed that AG interventions did not significantly alter foot pain upon reloading. Likewise, calcaneal bone demineralization has been reported by multiple authors with unloading, with microgravity analogues and after exposure to space (Armbrecht et al., 2010; Belavy et al., 2011; Beller et al., 2011; Gabel et al., 2022; Liu et al., 2023; Sibonga et al., 2007; Stavnichuk et al., 2020). The present trial results demonstrate that recreating AG during 30 min daily was inefficient at preventing calcaneal demineralization. Calcaneal BMAT up to 2 years after inactivity has not, to our knowledge, been reported. In this study, we found that calcaneal bone density recovery was incomplete 2 years after 60 days of bed rest.

The BMAT has multiple physiological functions locally, in the bone marrow, where it interacts with the bone and haemopoietic tissues (Hawkes & Mostoufi‐Moab, 2019). In addition, BMAT mediates systemic functions through adipokine production (Pachón‐Peña & Bredella, 2022; Piotrowska & Tarnowski, 2021). Although bone is mechanosensitive, it is unclear whether the mesenchyme‐derived BMAT also responds to decreased or increased mechanical forces. Multiple reports have identified BMAT upregulation with inactivity (Liu et al., 2021; Trudel et al., 2012, 2009). The precise mechanisms for BMAT modulation remain largely unknown. A bone marrow adipose switch posited that altered mechanical conditions would direct pluripotent mesenchymal cell differentiation towards the bone or the adipose phenotype; less mechanical stimulation would engage mesenchymal precursors to differentiate towards the adipocyte lineage, whereas mechanical stimulation would drive osteoblastic differentiation (Veldhuis‐Vlug & Rosen, 2017). Although the adipocyte switch mechanism is appealing to explain BMAT accumulation from lack of mechanical stimulation, it would not explain large reductions in BMAT after a few days of remobilization (Liu et al., 2021). Alternatively, BMAT modulation might be related to its role as a preferential local energy supply for haemopoietic and bone functions (Dirckx et al., 2019; Suchacki et al., 2020; Veldhuis‐Vlug & Rosen, 2018). The demonstration of bone marrow reconversion (decrease in BMAT) spatially and temporally associated with hypermetabolic bone and haemopoietic functions supported this possibility (Fairfield et al., 2021; Liu et al., 2021, 2023). Most experimental data were obtained by studying haemopoietically active bones. Investigating BMAT in the calcaneus, which is not involved in haemopoiesis, might contribute new elements regarding its modulation. In the present study, calcaneal BMAT was also unaffected by the unloading and inactivity of prolonged bed rest and interventions recreating weight‐bearing forces in the lower limbs. Upregulation of BMAT has been reported in various models of inactivity (Liu et al., 2021, 2023; Trudel et al., 2012, 2009). The absence of upregulation suggested a lack of calcaneal BMAT mechanosensitivity or of a significant decrease in energy supply for the bone. However, these findings might be blurred by a ceiling effect. The calcaneal BMAT at baseline in the present study was 91%, in comparison to 40%–50% in the lumbar vertebrae and 50%–60% in the pelvis (Beekman et al., 2022; Wen et al., 2023). Detecting a significant increase in BMAT above 91% might have been beyond the limits of the experimental design and MR sequences. Statistically significant increases in BMAT were found in subgroup analyses (cAG men at 31 days of bed rest and control men at 90 days of reambulation) but were minimal (+0.4% and +0.6%, respectively) and part of multiple comparisons.

Also in the present study, calcaneal BMAT was not downregulated at reambulation after prolonged bed rest, when the haemopoietic system is hypermetabolic to overcome anaemia caused by the microgravity analogue (Culliton et al., 2021). These findings support that, unlike haemopoietic bones, calcaneal BMAT was not solicited for enhanced haemopoieses at reambulation and that local haemopoiesis is important for BMAT downregulation; no haemopoiesis, no BMAT decrease. BMAT as an energy source for local bone anabolism in the calcaneus after prolonged bed rest appeared insufficient to lower the BMAT significantly.

BMAT and BMD have shown consistent inverse correlation across different bones and interventions (Muruganandan et al., 2018; Shen et al., 2007), at the origin of formulating the adipose switch mechanism (Steward & Kelly, 2015). The present trial confirmed systematic inverse correlations between BMAT and BMD across bed rest, reambulation, AG interventions and in both sexes. These results confirmed close interactions between the two co‐located tissues. This intriguing but consistent interaction between BMAT and bone remains incompletely understood.

Sex‐specific effects were identified. Men in the cAG and control groups showed increased calcaneal BMAT at 31 days of bed rest and at 90 days of reambulation, respectively, and all data from men showed decreased calcaneal BMD; neither bed rest nor AG protocols influenced calcaneal BMAT or BMD in women (Figure 3). The lack of statistically significant comparisons in women might be attributed to the lower statistical power for the subgroup of women (*n* = 8) than for the subgroup of men (*n* = 16). Larger sample sizes might allow a clearer determination of a sex‐specific response of calcaneal BMAT and BMD to unloading and AG interventions.

Foot pain after returning from space or in the bed rest space analogue can have multiple aetiologies, such as soft tissues (muscle atrophy, tendon/ligament enthesis remodelling) or altered motor control mechanisms occurring with unloading and reloading. The present new findings on BMAT and BMD support the initiation of future research to measure these key determinants of foot pain precisely.

These results are clinically relevant for multiple reasons. They demonstrate the immediate and long‐term deleterious effects of prolonged bed rest on the calcaneus BMC. They stress that interventions during inactivity are needed to prevent the bone loss and that rehabilitation protocols for return to activity need to target the lower limbs and treat the bone losses. In this study, a mechanical intervention of 30 min daily of AG did not prevent foot pain in most bedridden participants, and little research has focused on alleviating these symptoms. Finally, unloaded calcanei with decreased BMD were associated with more avulsion fractures as a mode of failure of the Achilles tendons (Matsumoto et al., 2003; Squires et al., 2001; Trudel et al., 2007; Wren et al., 2001). Current International Space Station exercise countermeasures do not protect astronauts fully from bone loss (Konda et al., 2019). Despite the introduction of the Advanced Resistive Exercise Device in combination with a treadmill and cycling ergometer, high‐magnitude moderate‐frequency loading was unable to rescue calcaneal bone demineralization (English et al., 2020). Our space analogue study using low‐magnitude moderate‐frequency loading also failed to rescue calcaneal bone loss in men. These findings emphasize the need for effective interventions to prevent and treat bone loss with unloading.

### 4.1 Limitations

This study has several limitations. Calcaneal BMAT and BMD were measured on the right foot, and changes related to footedness would have been missed. MRI and DXA administration were, at some time points, offset by a few days, which could have affected the correlation data. Measurements planned at reambulation day 365 were delayed to reambulation day 450 owing to the coronavirus disease 2019 pandemic. MR spectroscopic analyses could reveal changes in the calcaneal BMAT metabolic profile (Karampinos et al., 2018; Singhal et al., 2016). The study had an unequal number of men and women, preventing complete sex‐adjusted comparisons.

## 5 CONCLUSION

Prolonged antiorthostatic bed rest caused foot pain and decreased calcaneal BMD, which were not rescued by AG interventions. Calcaneal BMAT was neither modulated during bed rest nor at reambulation, possibly owing to a ceiling effect and demonstrating differences between haemopoietically active and inactive bones in adults. Decreased calcaneal BMD up to 2 years after bed rest has clinical implications for rehabilitation and the risk of avulsion fracture in patients returning to activity after prolonged bed rest or in astronauts returning from space.

## AUTHOR CONTRIBUTIONS

Conceptualization: Guy Trudel, Gabriele Armbrecht; Data curation: Guy Trudel, Tammy Liu, Gerd Melkus, Alain Berthiaume, Gabriele Armbrecht; Formal analysis: Guy Trudel, Tammy Liu, Gerd Melkus, Tim Ramsay; Funding acquisition: Guy Trudel, Gabriele Armbrecht; Investigation, Writing—original draft: Guy Trudel, Tammy Liu, Gerd Melkus; Visualization: Guy Trude, Tammy Liu; Writing—review & editing: Guy Trudel, Tammy Liu, Gerd Melkus, Tim Ramsay, Alain Berthiaume, Gabriele Armbrecht. All authors approved the final version of the manuscript and agree to be accountable for all aspects of the work in ensuring that questions related to the accuracy or integrity of any part of the work are appropriately investigated and resolved. All persons designated as authors qualify for authorship, and all those who qualify for authorship are listed.

## CONFLICT OF INTEREST

None declared.

## Data Availability

The data that support the findings of this study are available from the European Space Agency Science Data Center. Restrictions apply to the availability of these data, which were used under licence for this study.

## Inclusion and exclusion criteria

### Inclusion criteria

- Physically and mentally healthy test subjects who are able and declare their willingness to participate in the entire study and successfully passed the psychological and medical screening
- Aged between 24 and 55 years old, with a body mass index of 19–30 kg/m^2^, height between 153 and 190 cm
- Non‐smoker, for ≥6 months before the start of the study
- Capable of completing the study
- Willing to stay in bed, with or without weight‐bearing (AG), for 60 days
- Demonstrable medical insurance and official certificate of absence of criminal record
- Demonstrable dentist certificate

### Exclusion criteria

○ Drug, medication or alcohol abuse (regular consumption of >20–30 g alcohol/day)
○ A requirement for any prescription medications
○ Vegetarian, vegan (during the study)
○ Migraine, chronic headache
○ Insomnia or other sleep disorders
○ Previous psychiatric illness
○ Claustrophobia
○ Increased intraocular pressure
○ Any eye disorder that could significantly impact or jeopardize visual function
○ Hyperopia > +5.0 dioptres
○ Myopia > −6 dioptres
○ Astigmatism > 3 dioptres
○ History of laser surgery, glaucoma and retinal surgery
○ Hiatus hernia
○ Gastro‐oesophageal reflux
○ Gastrointestinal stenosis, dysphagia
○ Current or history of chronic bowel disease
○ Diabetes mellitus
○ Rheumatic illness
○ Current or a history of (chronic) pulmonary disease
○ Current muscle or joint disease or disorder
○ History of prolapsed intervertebral disc
○ Chronic back complaints
○ Bone fractures <1 year prior to study
○ Kidney disorder: deviations from normal values for creatinine in plasma. Deviations from normal values (normal values for creatinine in plasma < 1.20 mg/dl) and estimated glomerular filtration rate
○ History of kidney stones
○ History of (chronic) cystitis, hydronephrosis, pyelonephritis
○ Anaemia: haemoglobin under normal values. (Normal values of haemoglobin for men: 13.0–17.5 g/dl; women 12.0–16.0 g/dl)
○ Elevated risk of thrombosis
○ Pronounced orthostatic intolerance (<10 min standing and/or not able to withstand AG)
○ History of elevated intracranial pressure and associated central nervous disorders
○ Current or history of haemorrhagic diathesis or coagulation disorders
○ History of spinal cord disease, including radiculopathy, myelopathy or neuropathy
○ History of skull/cranial surgeries
○ History of adverse events to local anaesthesia
○ An abnormal androgen or oestrogen status (tested only upon speculation)
○ Female candidate is pregnant
○ Female candidate is on oral contraceptives or contraceptive patch up to 4–6 months prior to study start
○ Female candidate is in menopause or post‐menopause or is on hormone replacement therapy
○ Female subjects without a normal length menstrual cycle (20–36 days)
○ Inability or unwillingness to perform the required tests
○ A medical or orthopaedic condition that would preclude bed rest or exercise, as determined by the examining and overseeing physician of the bed rest study
○ Not within two standard deviations of normal bone mineral density (measured by DXA) for hip and lumbar spine based on *T*‐score (young adult‐peak bone mass, Caucasian, sex, but not age‐adjusted)
○ Metal implants (or objects like metallic slivers in the eyes, or bullets or shrapnel in the body) or other kinds of bone synthesis materials that are not well fixed; tattoos or permanent make‐up incompatible with MRI
○ Participation in a (clinical) study within the last 3 months before the start of this study that confounds participation in the AGBRESA study
○ Known history of vertigo, nystagmus, neurological conditions, vestibular or gait disorders
○ Previous heart surgery
○ History of cerebrovascular or brain disease, tumour, injury, surgery or malformation
○ Disorders of cerebrospinal fluid circulation (i.e., hydrocephalus, idiopathic intracranial hypertension)
○ Tinnitus; sensorineural hearing loss > 30 dB, or implanted hearing device
○ Known Chiari malformation
○ History of fracture(s) at proximal femur (hip area), <2 years and/or with remaining deficit and/or implants
○ Any other condition that makes the test subject unsuitable for study inclusion in the opinion of the project team
○ Fractures, metal implants of proximal femur
○ Pacemaker
○ Prior head surgery with metal implants
○ Other metal implants may or may not be a problem and need to be confirmed with a radiologist in each case
